# Preface

**DOI:** 10.2174/011570159X441819250909095415

**Published:** 2025-09-15

**Authors:** Ferdinando Nicoletti

**Affiliations:** 1 Department of physiology and Pharmacology, Sapienza University of Rome, Rome, Italy;; 2 Department of Molecular Neuropharmacology, IRCCS Neuromed, Pozzilli, Italy

I am pleased to announce that the Impact Factor (IF) for *Current Neuropharmacology* has increased to 5.3 in 2025. This is a remarkable achievement, especially at a time when the IFs of many journals have remained stable or even decreased. CN publishes review articles, research articles, and editorials focusing on basic neuroscience, neuropharmacology, translational research, and clinical studies across all fields of neuroscience. Leading experts in the fields of psychiatric disorders, Parkinson’s disease, and multiple sclerosis published their articles in CN in 2025, and many have upcoming articles scheduled for 2026. It is a great merit of the kind and professional associates of the Bentham team, who devote their best efforts to coordinating and continuously monitoring the editorial activities of the journal. I am fortunate to collaborate with many of them on a daily basis. I would like to personally appreciate all of them, and in particular Rabia Moin, with whom I began my tenure as an Editor-in-Chief (EIC) of CN years ago. Rabia Moin reflects the spirit of all members of the Bentham team with her kindness and high efficiency. I am impressed by the list of 27 special issues committed for 2026, which include topics, including *inter alia,* CNS inflammation, neuropharmacological aspects of brain cancers, new therapeutic strategies to achieve neuroprotection in neurodegenerative disorders and stroke, mitochondria as therapeutic targets in CNS disorders, the role of microbiota in neuropsychiatric disorders, the neurobiology of emotional disorders, neuropharmacology of cognition, learning and memory, the use of cannabis and cannabinoids in CNS disorders or drug addiction, the use of natural products in neurology and psychiatry, and pharmacological aspects of brain and spinal cord injury. I look forward to evaluating all relevant contributions with the invaluable assistance of the Editorial Board members and reviewers, who have performed excellent work throughout 2025. The updated list of keywords for CN will help attract an increasing number of contributions. My personal commitment for 2026 is to facilitate as much as possible the interaction between authors and the excellent collaborators of the Bentham team, thereby speeding up the publication time of accepted articles. It remains a great honor and pleasure for me to continue my role as an EIC of CN, with the hope of meeting expectations and contributing to the journal's success.

